# Richard Ho-Ping Sia: pioneer of infectiology in China

**DOI:** 10.1093/procel/pwac050

**Published:** 2022-11-04

**Authors:** Xudong Liu, Yuanmeng Li, Naishi Li

**Affiliations:** Medical Science Research Center, State Key Laboratory of Complex Severe and Rare Diseases, Peking Union Medical College Hospital, Chinese Academy of Medical Science and Peking Union Medical College, Beijing 100050, China; Department of Endocrinology, NHC Key Laboratory of Endocrinology, State Key Laboratory of Complex Severe and Rare Diseases, Peking Union Medical College Hospital, Chinese Academy of Medical Science and Peking Union Medical College, Beijing 100730, China; Department of Endocrinology, NHC Key Laboratory of Endocrinology, State Key Laboratory of Complex Severe and Rare Diseases, Peking Union Medical College Hospital, Chinese Academy of Medical Science and Peking Union Medical College, Beijing 100730, China; School of Humanity and Social Sciences, Chinese Academy of Medical Science and Peking Union Medical College, Beijing 100730, China

**Figure 1.**
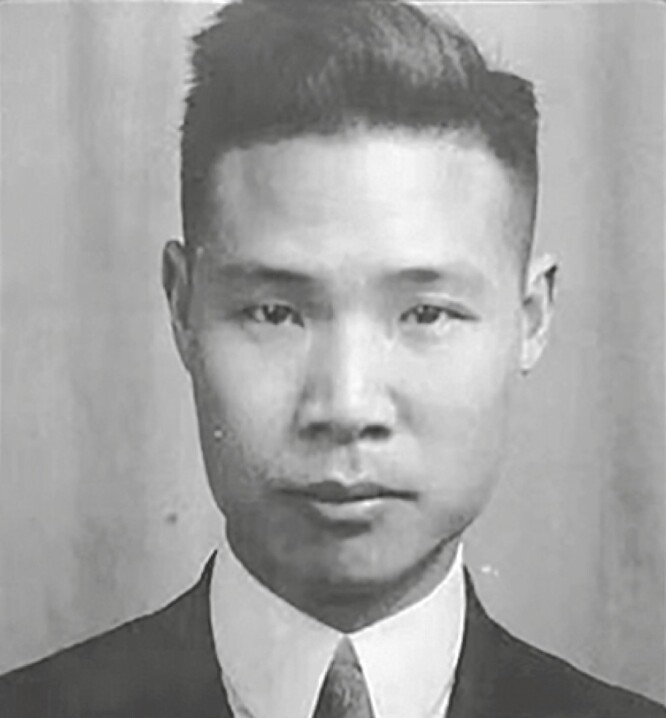
Richard Ho-Ping Sia.

Dr. Richard Ho-Ping Sia (谢和平) was born in Xiamen (Amoy) on 28 April 1895. After graduating from Boone University in Wuchang in 1914, he went to the Medical School of Western Reserve University in Cleveland, USA, to study clinical medicine, and received his MD degree in 1918. After completing 1-year clinical rotation training at Cleveland City Hospital, he returned to China in 1919 and joined Peking Union Medical College (PUMC) Hospital as an Assistant Resident. He was promoted to Assistant in 1920, and then worked as a physician treating infectious diseases in the Department of Medicine of PUMC Hospital. In 1939, he left PUMC for the University of Hawaii, where he served as a lecturer in bacteriology and school physician, and later practiced medicine in Honolulu until his retirement (Sia, 1971; Zhang and Li, 2016). Dr. Sia completed almost all of his academic career at PUMC. As he worked at PUMC Hospital for 20 years with a primary focus on infectious diseases, which were highly prevalent in China at that time. There is no doubt that he made significant contributions to the Department of Medicine of PUMC Hospital (Fig. 1).

PUMC was founded by the Chinese Medical Board and was entirely supported by the Rockefeller Foundation of the United States. The foundation stone laying ceremony of new PUMC buildings was held in 1917. It was the first medical school in China with an 8-year medical education program and an advanced nursing program. In 1919, Dr. Sia returned to China as an assistant resident in medicine at PUMC Hospital. From 1920 to 1921, Dr. Richard Ho-Ping Sia was the only Chinese doctor among the nine internal medicine staff according to the annual report of PUMC Hospital. He held the position of Assistant together with Otto Willner. Therefore, in a sense, Dr. Sia was one of the founders of the Department of Medicine, especially in the field of infectiology. The other seven were Professor Franklin C. McLean (President and Head of Medicine), Associate Professor Oswald H. Roberson (Head of Infectiology), Associate Professor Andrew H. Woods (Head of Neuropsychiatry), H. Jocelyn Smyly, Charles W. Young, John H. Korns, and William G. Lennox, the latter four of whom were all Associates.

In 1923, Prof. McLean resigned and returned to the USA, later serving as the Dean of the University of Chicago School of Medicine. As a consequence, Dr. Roberson took over his position. In 1926, Dr. Roberson was recruited to Chicago by Prof. McLean, at which point Francis R. Dieuaide took over as the director of Medicine. Meanwhile, Dr. Sia went to New York to collaborate with Oswald Avery from 1929 to 1930. After his return to PUMC, he was in charge of infectiology, and the attending physician of the infectious disease isolation ward until he left PUMC in 1939. His detailed comments on each patient in the ward were recorded in a large number of medical records. From November 1927 to August 1928, Dr. Sia also served as the head of the Medical Records Committee of PUMC. Therefore, Dr. Sia had undertaken a lot of clinical work in Internal Medicine, especially related to infectious diseases. By the time he left PUMC in 1939, Dr. Sia was the only physician in the entire Internal Medicine department who had worked for two decades. He had made remarkable contributions to the development of Internal Medicine at PUMC.

Dr. Sia devoted himself to the study of infectious diseases at the PUMC. He published two papers on kala-azar disease in 1921 (Sia, 1921; Sia and Wu, 1921), one of which was co-authored with Biochemist Wu Hsien (吴宪). In this collaborating work published in the *Chin Med J*, he described a method for the detection of kala-azar, later known as “Sia’s experiment.” Plasma was dropped into a test tube filled with demineralized water, and precipitation or flocculation indicated a positive reaction. This euglobulin test was considered to be the earliest screening method for primary macroglobulinemia. After discussion with Roberson, Sia concluded that the most common types of pneumococci in China were types I and IV.

**Figure 2.**
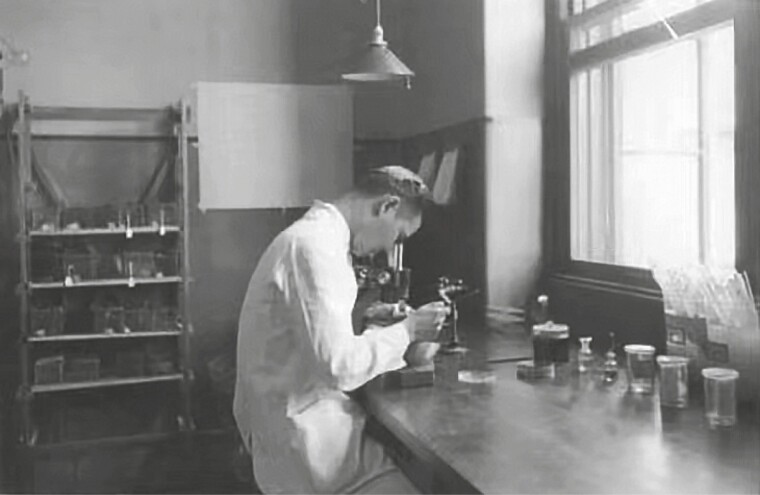
Dr. Sia in the infectious disease laboratory at PUMC.

From 1923 to 1927, Drs. Sia and Roberson designed a series of experiments for a study titled “Inhibition of the Growth of *Pneumococcus*” to systematically explore the scientific questions in this field. In this series of studies, they discovered the protective effect of gelatin, which extended the survival time of pneumococci in suspension at room temperature from a few hours to 6–7 days (Robertson et al., 1924). They also showed a strong ability to invent and create. To confirm the growth inhibition and bactericidal effect of a normal serum–leukocyte mixture on pneumococcus, they developed a new technique that most closely mimics the *in vivo* conditions in animals (Robertson and Sia, 1924a), which also included a method for keeping the serum–leukocyte mixture in constant motion. By exploring the effects of specific anti-pneumococcal sera and pneumococcal soluble substances on the growth of pneumococci in normal serum–leukocyte mixtures, Roberson and Sia found that the inhibitory and bactericidal effects of immune sera and leukocytes on pneumococci were quantitative in nature (Robertson and Sia, 1924b), whereas pneumococcal soluble substances can promote the growth of pneumococci to a certain extent (Sia, 1926), and it is highly specific to the type of pneumococcus. They also further explored the mechanism of the anti-pneumococcal effects of serum–leukocyte mixtures, and found that the difference in immunity of some mammals to pneumococcal infection was mainly due to the different concentrations of anti-pneumococcus opsonin in the blood (Robertson and Sia, 1927). Based on this series of experiments, they established several new experimental methods, examined problems related to the growth inhibition of pneumococci, and promoted the progress of pneumonia research (Fig. 2).

After this series of studies, Dr. Sia continued to investigate bacterial pneumonia. Based on animal experiments, the relationship between acquired humoral immunity and the recovery mechanism of human lobar pneumonia was further studied (Sia et al., 1928). During his stay in New York, Dr. Sia and two colleagues developed a new method for measuring specific precipitable proteins in anti-pneumococcal serum to demonstrate a quantitative relationship between specific precipitable proteins in the serum and immune protection (Heidelberger et al., 1930). This fast, simple, and low-cost method was recommended to replace the traditional mouse protection test.

Between 1929 and 1930, Martin H. Dawson and Richard Ho-Ping Sia experimented with *in vitro* transformation of pneumococci (Dawson and Sia, 1931; Sia and Dawson, 1931), which is considered the most important contribution in Dr. Sia’s academic life. The relationship between this research and pervious work at PUMC, as Rao (2014) pointed out, included at least the choice of animal serum (Dawson and Sia, 1931), because Sia found that the serum of various animals generally had anti-R properties (Sia, 1926). During their studies using horse serum, Sia and Dawson discovered that the converted substances were not polysaccharides (Sia and Dawson, 1931). “This experiment was one step in the chain of studies that showed that deoxyribonucleic acid is the transforming substance, and that DNA is the carrier of the genetic code.” The American Biochemist, A. Baird Hastings, described this work as “the classic but insufficiently recognized studies on which subsequent research that earned three Nobel Prizes was based.” (Bowers, 1971) Avery’s loss of the Nobel Prize was regrettable, but his contributions were world-renowned, whereby the research done by Richard Ho-Ping Sia and Martin H. Dawson, which constituted the foundation of Avery’s work, should not be neglected.

Dr. Sia worked at PUMC Hospital for 20 years, which was unique at the PUMC Department of Medicine prior to 1942. Many facts indicate that Dr. Sia was recognized by PUMC for his ability and achievements. In 1927, he was appointed as the head of the Medical Records Committee. In 1931, just 1 year after becoming assistant professor, he was promoted to associate professorship. Furthermore, in 1939, when Dr. Sia decided to leave, PUMC was still preparing to renew his employment for another 4 years. His main research work at PUMC focused on pneumococci, and he continued research in this field during his time as a visiting scholar at the Rockefeller institute of Medicine, where he devoted himself to *in vitro* transformation of pneumococci. Dr. Sia published nine papers in the *Journal of Experimental Medicine* (*J Exp Med*), which was a great academic achievement at the time (Rao, 2014). The academic platforms of PUMC and the Rockefeller institute of Medicine were world-class, which laid a good foundation for his scientific research. Additionally, *J Exp Med* was founded by the Johns Hopkins University School of Medicine, with William Welch as the first editor-in-chief. When the sponsor of the journal was moved to the Rockefeller institute of Medicine, the director of this academic establishment, Simon Flexner, served as the second editor-in-chief until 1935. William Welch and Simon Flexner were the most famous medical educators in North America at the time, and both were important medical scientists in the process of founding PUMC. This might explain why PUMC scholars like Dr. Sia preferred submitting to *J Exp Med*. As such, Dr. Sia is an important figure in the history of the Department of Medicine at PUMC. Further in-depth study of relevant data is of great significance to the history of infectiology in China.
